# The Changing Landscape of Breast Cancer: How Biology Drives Therapy

**DOI:** 10.3390/medicines3010002

**Published:** 2016-01-21

**Authors:** Sarah Friend, Melanie Royce

**Affiliations:** 1 Hematology Oncology, University of New Mexico Comprehensive Cancer Center, University of New Mexico, 1201 Camino de Salud N.E., MSC 07-4025, Albuquerque, NM 87131, USA; scfriend@salud.unm.edu; 2 New Mexico Cancer Care Alliance, UNM Comprehensive Cancer Center Multidisciplinary Breast Cancer Clinic &Program, University of New Mexico, 1201 Camino de Salud N.E., MSC 07-4025, Albuquerque, NM 87131, USA

**Keywords:** breast cancer, targeted therapy, PI3K/AKT/mTOR Inhibitors, cyclin-dependent kinase (CDK) inhibitors, pertuzumab, Ado-trastruzumab-emtansine, angiogenesis inhibitors, poly (ADP-ribose) polymerases (PARP) inhibitors

## Abstract

Breast cancer is the most prevalent life-threatening cancer in women. Optimizing therapy to increase cure rates in early stage disease, and improving life expectancy and palliation for advanced stages, are goals driving major areas of research. The armamentarium of targeted treatments for breast cancer is ever expanding as understanding of breast cancer biology deepens. A revolution in our treatment was heralded a decade ago by the introduction of trastuzumab for human epidermal receptor-2 positive (HER2+) disease resulting in remarkable reductions in recurrence and improvements in overall survival (OS). Advances continue to be made in other breast cancer subtypes targeting key activating pathways for therapeutic development. However, for these other targeted agents, improvement in OS has been elusive. This article focuses on the development of targeted therapy in breast cancer focusing primarily on the last 5 years, to illustrate that as we understand the complex pathways allowing the dysregulated cell to become malignant, it also propels us closer towards the promise of precision and personalized medicine.

## 1. Introduction

**Figure 1:**
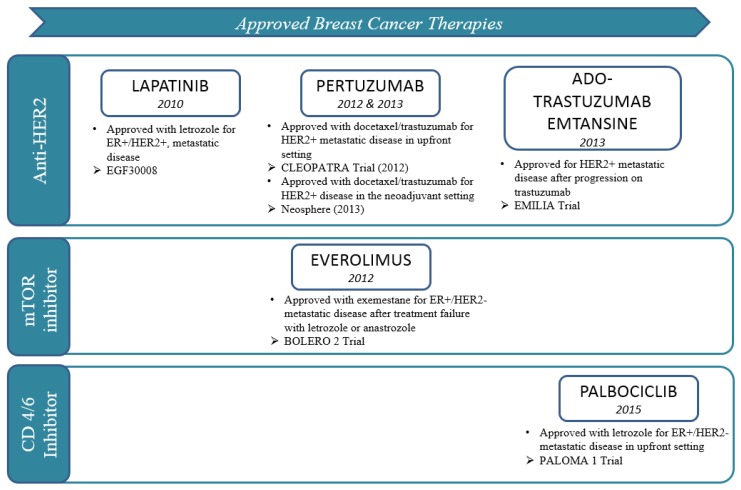
Unites States Federal Drug Administration (U.S. FDA)-approved drugs (since 2010).

In the last 5 years, advances in targeted therapy for other solid malignancies such as melanoma and lung cancer may have overshadowed what has been accomplished in breast cancer. However, breast cancer has leveraged the advantages of targeted therapy long before the modern era of targeted therapy. Before the concept of targeted therapy was conceived, it was being tried for the management of breast cancer. In 1896, Sir George Thomas Beatson published on bilateral oophorectomy, a form of estrogen receptor (ER) pathway targeted therapy, to treat inoperable breast cancer. Targeting the ER pathway is now standard practice for hormone receptor positive (HR+) breast cancer, often accomplished with drugs including tamoxifen, aromatase inhibitors (AI), and fulvestrant. Another landmark targeted therapy for breast cancer was trastuzumab, targeting the human epidermal receptor-2 (HER2) signaling pathway. With the approval of trastuzumab for the adjuvant treatment of early-staged HER2 positive breast cancer, wide availability of this agent changed the natural history of the disease, leading to fewer recurrences and more cures. Unfortunately, such landmark events have been infrequent with only incremental advances being made reflected primarily in improvements in the surrogate endpoint of progression free survival (PFS) rather than overall survival (OS) in most of the targeted therapies developed for other breast cancer sub-types, with the caveat that there is often an inverse correlation between these endpoints depending on duration of post progression survival (PPS) [1,2]. Nonetheless, these novel targeted agents have provided control of disease for some patients and palliation for many more. (U.S. FDA) approved targeted agents (Figure 1), will be discussed in detail in this review along with how they may be integrated in the current treatment paradigm(s) of breast cancer. Promising targeted agents will be highlighted albeit in a more abridged fashion, and issues to be vigilant about as we develop targeted therapies for the future will be discussed.

## 2. PI3K/AKT/mTOR Pathway: Everolimus, a Paradigm for Overcoming Endocrine Resistance

At time of progression, downstream signal transduction pathways have often become upregulated to serve as an escape route from estrogen receptor (ER) blockade. One such pathway is the intracellular phosphoinositide 3-kinase (PI3K)/protein kinase B (AKT)/mammalian target of rapamycin (mTOR) pathway [3]. When proteins within this downstream pathway become activated, cellular proliferation and survival of breast cancer cells ensues, leading to endocrine resistance [4]. In the metastatic setting, hormone receptor positive (HR+) tumors inevitably progress despite initially responding to hormonal treatments, a phenomenon described as acquired resistance. Because HR+ breast cancers still comprise the vast majority of all breast cancer sub-types, resistance to endocrine therapy remains an important clinical problem. Extensive research has been devoted to finding targeted agents to overcome endocrine resistance by looking into the various escape mechanisms from endocrine blockade and looking into ways of improving the efficacy of current hormonal therapies.

Knowledge of the PI3K/AKT/mTOR pathway has led to the development of powerful mTOR inhibitors, such as everolimus, which was approved in 2012 for the treatment of ER+/HER2-metastatic breast cancer after progression on prior non-steroidal aromatase inhibitor (AI). In the pivotal phase III BOLERO-2 trial, everolimus with exemestane more than doubled PFS compared with exemestane, from 3.2 months to 7.8 months (hazard ratio (HR) 0.45; 95% CI, 0.38 to 0.54; exploratory *p* < 0.0001) [5]. OS data, however, did not significantly reduce the risk of death in patients treated with everolimus and exemestane compared to exemestane and placebo [6]. Several hypotheses exist to explain the inconsistency of a significant PFS benefit without significant effects on OS, such as an imbalance in post-study salvage therapy between the treatment arms or too small a sample size to detect a small difference in OS since it was a secondary endpoint. However, one should consider another possible hypothetical explanation based on tumor biology. When the mTOR complex 1 (mTORC-1) is inhibited, a negative intracellular feedback loop between mTORC-1 and the IGF-1 signaling axis is released, paradoxically activating AKT [6]. Thus, the tumor activated at time of progression may be more aggressive; thus, despite the initial delay in progression, the cancer may be ultimately harder to treat on subsequent progression. Whether this laboratory observation is clinically relevant remains to be proven.

The TAMRAD phase II trial of tamoxifen plus everolimus had an inclusion criteria similar to BOLERO-2, but the primary end point of the two studies were different, with the primary end point of TAMRAD being clinical benefit rate, defined as the percentage of all patients with a complete or partial response or stable disease at 6 months [7]. There was a 55% reduction in the risk of death associated with tamoxifen plus everolimus (HR 0.45; 95% CI, 0.24 to 0.81; exploratory *p* = 0.007). The benefit of everolimus in AI-resistant metastatic breast cancer was suggested, but long-term data was unavailable. More clinically meaningful results can be taken from BOLERO-2 compared to TAMRAD because the former was a larger, phase III trial with more relevant endpoints. What may prove most useful from TAMRAD, however, was the identification of biomarkers to determine which patients are most likely to derive the most benefit. There was a positive correlation between late effectors of mTORC-1 activation, a positive correlation between AKT-independent mTORC-1 activation, and an inverse correlation between canonical PI3K/AKT/mTOR pathway and everolimus efficacy [8]. It should be emphasized, however, that this observation has not been validated and is not ready to be utilized in clinical practice.

The clinical development of mTOR inhibitors, has not been as straightforward and at times has been perplexing. Results from the phase III randomized front-line HORIZON study contrasted those from BOLERO-2. The study design was different between the two trials, with HORIZON using a different mTOR inhibitor and enrolling patients in the front-line setting. In that trial, the combination tested was that of temsirolimus with letrozole *vs.* letrozole/placebo in over 1100 patients withuntreated ER+/HER− metastatic disease. The study was terminated early for futility with no improvement in PFS (median, 9 months; HR, 0.90; 95% CI, 0.76 to 1.07; *p* = 0.25). More grade 3 to 4 events (37% *vs.* 24%) were experienced with the combination therapy [9]. Several postulates have been made as to why one mTOR inhibitor works while not another have been made, but the exact reasons for this observation is unclear. Whether an mTOR inhibitor should only be combined with endocrine therapy to restore sensitivity or whether use in the front line will prevent or delay resistance remains unanswered. BOLERO-4, and other trials of everolimus in the earlier-stage setting may provide answers, but we will have to wait and see.

Making things more perplexing is that a similar strategy has not been as clinically meaningful for a different subtype of breast cancer such as HER2+ disease. As investigated in the BOLERO 3 trial of vinorelbine plus everolimus *vs.* vinorelbine/placebo in trastuzumab pre-treated patients, the statistically significant improvement in PFS is short, 7.00 months (95% CI 6.74–8.18) with everolimus *vs.* 5.78 months (5.49–6.90) with placebo (HR 0.78 (95% CI 0.95); *p* = 0.0067) and come at the price of increased toxicity [10]. Furthermore, the subsequent BOLERO 1 trial in previously untreated patients (paclitaxel plus everolimus *vs.* paclitaxel/placebo in HER2+ metastatic breast cancer) had a median PFS that was virtually identical in the two treatment arms, 14.95 months with addition of everolimus *vs.* 14.49 months with placebo [11]. The subgroup analysis showed that there was a 7.2 month prolongation of PFS in the subgroup of HR−/HER2+ patients, although this did not meet the prespecified criterial for significance [11]. Without blocking the estrogen receptor in ER+ disease, the estrogen receptor provides an escape pathway leading to cell survival. In that way, the estrogen receptor activation provides alternative signals for proliferation in HER2+ disease. Newer studies will focus on blocking all known targetable pathways, including combinations of everolimus, letrozole, and trastuzumab. Given the side effect profile of everolimus, with mucositis, diarrhea, and anemia in greater than 30%, mTOR inhibitors with better tolerability will certainly be welcomed addition to our armamentarium. But more importantly, biomarkers that are predictive of benefit from mTOR inhibition is a true unmet need. Indeed this is true for many of the targeted therapies being developed today. Valiant search has certainly been attempted through the translational sub-studies of the aforementioned trials [8]. Unfortunately, although some candidates have been identified, none have panned out to be clinically useful for routine use at present time.

The role of mTOR inhibitors in the clinical arena to date in breast cancer outside of a clinical trial is to extend the mileage out of the endocrine therapy by overcoming secondary resistance to prior ER blockade. Survival impact is not expected, but this strategy of dual pathway inhibition targeting ER and mTOR can delay the need for switching to chemotherapy, which can be quite an attractive option for many patients.

## 3. Cell Cycle Regulatory Machinery: Palbociclib, a Paradigm for Synergy with Endocrine Therapy

Uncontrolled cellular proliferation is a hallmark of malignancy. Understanding what controls the tightly regulated proliferative process and what disrupts it to cause unrestrained growth is fertile ground for development of targeted therapies for breast cancer. Several cell-cycle checkpoint proteins control progression through cell division from G1/S through M-phase including cyclin-dependent kinase (CDK), checkpoint kinase, WEE1 kinase, aurora kinase and polo-like kinase [12]. Among these proteins, those targeted against the cyclin-dependent kinases, (*i.e*., CDK inhibitors) are the most advanced therapeutics for breast cancer. The CDKs are a large family of serine threonine kinases that help control progression through the cell cycle by regulating phosphorylation [13,14]. For instance, CDK 4/6 and cyclin D regulate the G1/S transition through regulation of the retinoblastoma (RB) oncoprotein. When RB is phosphorylated, transcription factors are released allowing the cell to transition from G1 to S phase [15]. Inhibitors of CDK 4/6, therefore, keep RB in the unphosphorylated state and transcription factors remain bound to it, ultimately resulting in G1 arrest.

Palbociclib is the first-in-class, oral, reversible, highly selective inhibitor of CDK4/6 that has been approved for front-line treatment of metastatic ER+/HER2-breast cancer in combination with an AI (Figure 1). It received “Breakthrough Therapy” designation from the FDA after the open-label phase II PALOMA-1 trial demonstrated a statistically significant improvement in PFS when palbociclib was added to letrozole in the treatment of postmenopausal women with metastatic ER+/HER2-breast cancer who had not previously received any systemic treatment for their advanced disease [15]. This trial enrolled 165 patients, 43% had received chemotherapy and 33% had received endocrine therapy in the adjuvant/neoadjuvant setting. With a median follow-up of approximately 30 months for the palbociclib plus letrozole group and 28 months for the letrozole alone group, the median PFS was 20.2 months (95% CI 13.8–27.5) and 10.2 months (95% CI 5.7–12.6), respectively, (HR 0.488, 95% CI 0.319–0.748; one-sided *p* = 0.0004). The follow-up PALOMA-3 study confirmed the activity of palbociclib in this disease with a statistically longer PFS (HR, 0.42; 95% CI, 0.32–0.56; *p* < 0.001) seen among patients treated with fulvestrant plus palbociclib *vs.* fulvestrant alone in patients with HR+/HER2-metastatic breast cancer who had progression of disease during prior endocrine therapy irrespective of menopausal status [15]. OS data for the trial is premature at the time of publication. Palbociclib’s primary side effect is myelosuppression, with neutropenia being most pronounced (62.0% *vs.* 0.6%, grade 3 or 4 in the palbociclib/fulvestrant *vs.* placebo/fulvestrant combination, respectively). However, neutropenic fever was low at 0.6% in both treated groups. Additional studies testing palbociclib in various disease settings are ongoing. Other CDK 4/6 inhibitors such as ribociclib (LEE011) and abemaciclib (LY2835219) are also in advanced stages of therapeutic development (Table 1 [16]). While it remains to be seen what additional advantage(s) they offer beyond that of palbociclib, they will be welcomed additions to the treatment armamentarium for breast cancer because CDK 4/6 inhibitors could set the stage for how we may possibly approach the problem of primary resistance to endocrine therapy. Furthermore, it would be quite interesting to find out if the other cell-cycle checkpoint proteins will prove to be as good a target for therapeutic development. If so, time will tell if they will be efficacious as targeted agents and how much further they can propel us to a cure.

**Table 1:** Clinical Trials with cell cycle inhibitors in breast cancer [16].

**Trials with CDK 4/5 Inhibitors**					
**Study Name/Identifier**	**Drug(s)/Novel Agent(s)**	**Study Phase**	**N**	**Primary Endpoint**	**Disease Setting**
**PALOMA-2** NCT01740427	Letrozole +/− Palbociclib	II	650	PFS	Front-line, advanced/metastatic
**PENELOPE-B** NCT1864746	Palbociclib + Endocrine	III	800	DFS	Residual disease after neoadjuvant
**PEARL** NCT02028507	Palbociclib + exemestane *vs*. capecitabine	III	348	PFS	Metastatic after progression on AI
**neoMONARCH** NCT02441946	Abemaciclib + Anastrozole *vs*. abemaciclib *vs*. anastrozole	II	220	Δ Ki-67 at 2 weeks	Neoadjuvant, Stage I-III
**MONARCH** NCT2107703	Fulvestrant +/− Abemaciclib	III	630	PFS	Front-line, advanced/metastatic
**MONALEESA-3** NCT02422615	Fulvestrant +/− Ribociclib	III	660	PFS	1^st^ or 2^nd^ line, advanced/metastatic
**PALLAS** NCT02513394	Endocrine +/− Palbociclib	III	4600	DFS	Adjuvant, Stage II-III
**Trials with CDK aurora kinase inhibitors**					
**Study Name/Identifier**	**Drug(s)/Novel Agent(s)**	**Study Phase**	**N**	**Primary Endpoint**	**Disease Setting**
**2076-CL-005** NCT01639248	ENMD-2076	II	37	CBR	Metastatic TNBC
**13-033** NCT02187991	Paclitaxel +/− Alisertib	II	252	TTP	ER+ or −/HER2−, locally recurrent or metastatic

Abbreviations: Δ = change; CBR = clinical benefit rate; DFS = disease free survival; N = number of patients; PFS = progression free survival; TNBC = triple negative breast cancer; TTP = time to progression, CDK = cyclin dependent kinase, AI = aromatase inhibitor, ENMD-2076 = unnamed novel targeted agent.

## 4. Antibodies beyond Trastuzumab: Pertuzumab and Ado-Trastruzumab-Emtansine (T-DM1)

A key observation many decades ago was that overexpression of the HER2 protein and/or HER2 gene amplification confers a poorer prognosis in breast cancer compared to normal HER2 expression. Approximately 20% of breast cancers are HER2+, with the poor prognosis being mitigated by the advent of targeted therapy blocking the HER2 receptor. Despite the profound effects of trastuzumab, it took almost 15 years before another HER2 targeted antibody successfully gained FDA approval for clinical use. In 2012, pertuzumab, a humanized monoclonal antibody directed against the dimerization domain II of HER2 [17], was approved for the treatment of advanced disease in combination with chemotherapy and trastuzumab. The following year it received accelerated approval for use in the neoadjuvant setting. Within the heels of pertuzumab’s approval, T-DM1, an antibody-drug conjugate of trastuzumab and a cytotoxic drug derivative of maytansine [18], was approved for the treatment of HER2+ disease after progression on trastuzumab in the metastatic setting (Figure 1). Availability of these targeted therapies obviously expanded the therapeutic options of patients with HER2+ breast cancer, with an inadvertent (although not surprising) effect of shifting the treatment paradigm. Novel anti-HER2 antibodies have supplanted lapatinib, the only FDA approved targeted tyrosine kinase inhibitor (TKI) for the treatment of HER2+ metastatic breast cancer, moving the oral TKI down the line as a treatment option in this disease. The preferential use of the HER2-directed antibodies over lapatinib is perhaps to be expected given the impressive results from the EMILIA [19] and CLEOPATRA [20] trials showing statistically significant improvements in both PFS and OS. In contrast, the EGF100151 [21] study of lapatinib with capecitabine *vs.* capecitabine alone has at best only shown a trend in survival with the combination arm. To be fair, it must be pointed out that these are cross trial comparisons, but are being made here only as a potential explanation as to why a clinician might prefer to use the antibodies over the oral TKI, despite the convenience of oral therapy.

Biologically, why might a HER2-directed antibody be more effective than an oral TKI, especially one like lapatinib which doubly targets both HER2 and the epidermal growth factor receptor (EGFR)? Perhaps it is the less often described immune-mediated effects of the HER2-directed antibodies that provide them their advantage. Both pertuzumab and trastuzumab (and by default T-DM1) are IgG1 antibodies, and therefore can induce antibody-dependent cell-mediated cytotoxic (ADCC) effects on HER2 overexpressing tumors [22]. Immunotherapy is now a well-accepted treatment for many solid tumors and even though no specific immunotherapy has been approved to date for breast cancer it is being actively explored (Table 2 [16]). Immunotherapy as a form of targeted therapy will not be discussed in this review as it merits a separate discussion. Readers are referred to independent reviews on this topic.

**Table 2:** Clinical Trials of Immunotherapy in Breast Cancer [16].

**Immune Checkpoint Inhibitors**					
**Study Name/NCT Identifier**	**Drug(s)/Novel Agent(s)**	**Study Phase**	**N**	**Primary Endpoint**	**Disease Setting**
**TONIC** NCT02499367	Nivolumab	II	84	PFS	TNBC, ≥2^nd^-line Metastatic
**4147523** NCT02395627	Pembroluzimab	II	58	ORR	Postmenopausal ER+, ≥2^nd^-line Metastatic
**Vaccine, Small Molecule Inhibitors & Others**					
**Study Name/Identifier**	**Drug(s)/Novel Agent(s)**	**Study Phase**	**N**	**Primary Endpoint**	**Disease Setting**
**11-202** NCT01570036	NeuVax	II	300	DFS	HER2+, Adjuvant
**OSU 13117** NCT01964924	Trametinib + GSK2141795	II	41	ORR	TNBC, ≥2^nd^-line Metastatic
**NYU 11-00598** NCT01421017	Imiquimod	I/II	55	ORR	≥2^nd^-line; + skin lesion, advance/metastatic

Abbreviations: DFS = disease free survival; N = number of patients; ORR = overall response rate; PFS = progression free survival; TNBC = triple negative breast cancer, HER2+ = human epidermal growth factor receptor positive, NCT = National Clinical Trials, OSU 13117 = unnamed novel targeted agent, NYU 11-00598 = unnamed novel targeted agent, TONIC = abbreviation for clinical trial name.

The approval of pertuzumab in the front-line treatment of advanced HER2+ breast cancer was established in the phase III CLEOPATRA trial which randomized approximately 800 women to either pertuzumab, trastuzumab, and docetaxel (THP) *vs.* placebo, trastuzumab, and docetaxel (TH) [20]. The pertuzumab–containing arm significantly prolonged PFS by 6.3 months compared to control (HR 0.86, 95% CI 0.58–0.80, *p* < 0.001) without any increase in cardiac toxic effects. Median OS was 56.5 months (HR = 0.68, 95% CI 0.56–0.84; *p* = 0.0002), or 15.7 month longer than control [20]. Similarly impressive results were seen in the neoadjuvant setting for the NeoSPHERE study where THP produced a 45.8% pathologic complete response (pCR) rate (95% CI, 36.1–55.7, *p* = 0.0141) compared to TH [23]. Importantly, approximately 17% of patients had a pCR with only dual anti-HER2 antibody therapy (TP) without chemotherapy, presenting an attractive option for women who cannot receive cytotoxic chemotherapy. This option, however, should be used very selectively, as a third of patients did not respond with just dual anti-HER2 therapy, indicating the need for additional treatment. It is worth keeping in mind that in omitting chemotherapy, we also give up the synergistic effects between trastuzumab and chemotherapy.

T-DM1 was developed as another strategy to target HER2 overexpressing breast cancers using a novel mechanism of action. The trastuzumab antibody and cytotoxic agent, emtansine, are conjugated by a stable linker such that delivery of the cytotoxic drug is targeted to HER2 overexpressing cells and minimizing exposure of normal tissues, resulting in improved therapeutic index [18]. The FDA approval of T-DM1 in patients with HER2+ metastatic breast cancer after progression on trastuzumab was based on the results of the EMILIA phase III trial which randomized 980 patients to either T-DM1 *vs.* lapatinib plus capecitabine [19]. Median PFS was 9.6 *vs.* 6.4 months, respectively, translating to a 3.2 months median prolongation in PFS with T-DM1 (HR 0.65, 95% CI 0.55–0.77, *p* < 0.001). Median OS was also improved for T-DM1 at 30.9 months *vs.* 25.1 months for lapatinib plus capecitabine (HR 0.68, 95% CI 0.55–0.85, *p* < 0.001). Cardiac toxicities for T-DM1 were similar to those expected for trastuzumab. Elevations in AST/ALT and thrombocytopenia were the only other notable grade 3 or 4 toxicities reported for T-DM1; these were manageable with dose modifications [19].

In just the last 5 years, treatment options for HER2+ breast cancer have increased. Improvements in both PFS and OS are expected to translate to better outcomes for many patients with this breast cancer subtype. More importantly, further cures are anticipated if the surrogate endpoint of pCR translates into the more tangible endpoints of reductions in risks of recurrence and overall survival in the adjuvant setting. Results from confirmatory trials like APHINITY [16], which compared adjuvant chemotherapy plus trastuzumab with or without pertuzumab, are eagerly awaited. Until those results are reported on, many patients fortunately will have access these novel anti-HER2 antibodies, including pertuzumab for adjuvant therapy through the accelerated approval process. More importantly, we have a biomarker we can assay in the tumor for response to anti-HER2 therapy. Unfortunately, this is not always the case for many of the evolving targeted agents.

## 5. Challenges and Pitfalls Developing Targeted Agents for Breast Cancer: Lessons from Bevacizumab and Iniparib

### 5.1. An Almost Failed Attempt Targeting Angiogenesis with Bevacizumab

Angiogenesis is the process of creating new blood vessels that occurs in both normal and cancerous tissue. The idea of targeting angiogenesis to prevent tumor proliferation appears rational and the first drug developed in this class for breast cancer was bevacizumab, a monoclonal antibody that binds to vascular endothelial growth factor (VEGF) A ligand, preventing its interaction with VEGF receptors (VEGFRs) on the surface of endothelial cells [24]. The clinical development of bevacizumab in breast cancer began with great expectation with the observation from the ECOG E2100 trial that it prolongs median PFS in metastatic disease [25]; however, there was no advantage seen in median OS [25]. Subsequent trials in the metastatic setting also failed to show a survival advantage and bevacizumab’s initial accelerated approval in breast cancer was rescinded by the FDA. In the non-metastatic setting, studies with bevacizumab initially showed improvement in the surrogate endpoint of pCR when combined with neoadjuvant chemotherapy [26], but subsequent adjuvant trials were also essentially negative [27,28]. It was not until the recent publication of NSABP B-40, a phase III randomized clinical trial of almost 1200 patients with early stage HER2-breast cancer, did we see a positive signal in OS. This trial had a 3 × 2 factorial design, where patients received standard chemotherapy with or without either capecitabine or gemcitabine given with or without bevacizumab in the neoadjuvant setting. Following surgery, those randomized to bevacizumab neoadjuvantly received an additional 10 cycles post-operatively. Study results showed that the addition of bevacizumab significantly increased median OS (HR 0.65, 95% CI 0.49–0.88; *p* = 0.004) but did not significantly reduce disease-free survival (HR 0.80, 95% CI 0.63–1.01; *p* = 0.06) [26]. Interestingly, the best benefit in the NSABP B-40 trial was seen in patients with HR+ tumors [26]. In contrast, the neoadjuvant GeparQuinto trial observed a pronounced effect in the triple-negative subgroup with a strong association between single nucleotide polymorphisms (SNPs) and a clinical pCR with bevacizumab [29]. Several differences exist between these two trials and whether those would be sufficient to biologically account for the differences observed is up for debate. The reality is that the positive results of NSABP B-40 is likely to make little impact on current treatment paradigm given the lack of a biomarker to know who really benefits from bevacizumab (or anti-angiogenic therapy in general) in light of the high cost and unique toxicities of this agent. What NSABP B-40 will likely spur is more dialogue on where we will even position anti-angiogenic agents in the breast cancer therapeutic armamentarium given the preponderance of negative trials (Table 3 [16]) and how we move forward henceforth to rationally develop novel anti-angiogenic agents in breast cancer.

**Table 3:** Trials with Angiogenesis Inhibitors for Breast Cancer [16].

**TKI with Anti-Angiogenic Properties**					
**Study Name/NCT Identifier**	**Drug(s)/Novel Agent(s)**	**Study Phase**	**N**	**Primary Endpoint**	**Study Outcome**
**SCRI BRE 122** NCT00887575	Neoadjuvant sunitinib + paclitaxel/carboplatin	I/II	54	pCR	Combo not recommended
**ZACFAST** NCT00752986	Fulvestrant +/− vandetanib	II	41	EFS	Terminated
**NSABP FB-6** NCT00849472	Neoadjuvant AC ➔ +/− pazopanib	II	101	pCR	Increased toxicity; combo not recommended
**A4061010** NCT00076024	Docetaxel +/− Axitinib	I/II	174	TTP	Not significant
**RESILIENCE** NCT01234337	Capecitabine +/− sorafenib	III	519	PFS	No advantage
**Monoclonal Antibody**					
**Study Name/NCT Identifier**	**Drug(s)/Novel Agent(s)**	**Study Phase**	**N**	**Primary Endpoint**	**Study Outcome**
**Rose/TRIO-12** NCT00703326	Docetaxel +/− ramucirumab	III	1144	PFS	No OS advantage

Abbreviations: TKI = tyrosine kinase inhibitor, NCT = National Clinical Trials, EFS = event free survival; N = number of patients; OS = overall survival; pCR = complete pathologic response; PFS = progression free survival; TTP = time to progression.

### 5.2. Missing the Mark with the PARP Inhibitor, Iniparib

The poly (ADP-ribose) polymerases (PARP) enzyme synthesizes ADP-ribose polymers that repair endogenous DNA damage leading to cell viability. Patients with *BRCA 1* or *BRCA 2* mutations have a deficiency of DNA repair by homologous recombination. Inhibition of PARP has been advanced as a unique targeted therapy for cancers harboring *BRCA 1/2* mutation as it leads to the accumulation of single strand breaks that are converted to double strand breaks that cannot be repaired by homologous recombination, which induces apoptosis [30]. The strong biological rationale that combining DNA damaging agents with PARP inhibitors leads to synthetic lethality in tumors harboring *BRCA 1/2* mutation was tested in breast cancer with the PARP inhibitor, iniparib, with initial promising results. The PrECOG 0105 trial was a single-arm phase II neoadjuvant study of gemcitabine and carboplatin, for early stage breast cancers with a primary end point of pCR [31]. Among 80 patients, the overall pCR rate was 36% (90% CI, 27–46). In patients with wild-type *BRCA 1/2*, the pCR was 33%, while among *BRCA 1/2* mutation carriers, the pCR rate was 47%; in the subset of *BRCA 1/2* mutation carriers with triple negative breast cancer (TNBC), pCR was 56%. Tumor genomic testing revealed a favorable pathologic response in both *BRCA 1/2* mutation in addition to sporadic TNBC with an elevated loss of heterozygosity (HRD-LOH) score [31]. Iniparib was subsequently investigated in using the same chemotherapy combination in metastatic TNBC. The initial phase II trial produced much excitement, showing that the addition of iniparib prolonged the median PFS from 3.6 months to 5.9 months (HR 0.59; *p* = 0.01) and the median OS from 7.7 months to 12.3 months (HR, 0.57; *p* = 0.01) [32]. However, the phase III confirmatory trial with iniparib in combination with gemcitabine plus carboplatin in metastatic TNBC was disappointingly negative, failing to meet the pre-specified criteria for the trial endpoints of PFS and OS [33]. Explanations abound for these negative results including the assertions that iniparib was not a bona fide PARP inhibitor. Ultimately, what can be said is that PARP inhibitors have not advanced as far ahead as initial expectations would have predicted, especially in the niche area of TNBC where there is no targeted therapy available to date. A meta-analysis of the PARP inhibitors showed an overall improvement of PFS but not OS and side effects are minimal, mainly neutropenia and asthenia, suggesting that as a class these drugs are well tolerated [34]. Clinical development of PARP inhibitors continues but more narrowly in *BRCA* mutation carriers rather than broadly in TNBC (Table 4 [16]). What has been highlighted by iniparib’s development is the importance of understanding the nuances of the biologic process we intend to impact and the need to use validated preclinical models to provide clarity as to how we are altering complex biologic systems with our targeted therapies so that we strike the right balance between taking targeted agents rapidly but not prematurely into the clinical arena.

**Table 4:** Trials of poly (ADP-ribose) polymerase (PARP) inhibitors [16].

**Trials Focused Primarily in Breast Cancer with Brca Mutation**					
**Study Name/NCT Identifier**	**Drug(s)/Novel Agent(s)**	**Study Phase**	**N**	**Primary Endpoint**	**Disease Setting**
**EMBRACA Study** NCT01945775	Talazoparib *vs*. Physician’s Choice	III	429	PFS	Metastatic breast cancer patients with *BRCA* mutation
**2014-0045** NCT02282345	Talazoparib	II	20	Toxicity, safety	Neoadjuvant, +*BRCA* mutation
**Trials in Breast (with or without *BRCA* Mutation) and other Malignancies**					
**Study Name/NCT Identifier**	**Drug(s)/Novel Agent(s)**	**Study Phase**	**N**	**Primary Endpoint**	**Disease Setting**
**OlympiA** NCT02032823	Olaparib	III	1320	DFS	Adjuvant, TNBC in high risk *BRCA 1/2*
**ComPAKT** NCT02338622	Olaparib + AKT inhibitor (AZD5363)	I	58	Safety, tolerability	Advanced solid tumors, *BRCA 1/2* mutation, TNBC or hyperactive PI3K-AKT pathway

Abbreviations: NCT = National Clinical Trials, DFS = disease free survival; N = number of patients; PFS = progression free survival; TNBC = triple negative breast cancer, AKT = abbreviation for proto-oncogene also known as protein kinase B or PKB, PI3K-AKT = phosphoinositide 3-kinase/AKT signaling pathway PI3K.

## 6. The Future of Novel Targeted Therapies: The Promise of Great Hope for Our Patients

The goal in treating metastatic breast cancer is to provide additional treatment options in order to improve symptom burden, quality of life and survival of patients with advanced disease. For early stage-disease, the goal is for a cure with the least toxic therapy. Although there was great promise that targeted therapies would bring less toxicity, the reality is that they bring different not necessarily less toxicity for patients. It remains a desirable goal to find less-toxic targeted agents, even better if they are novel, and efforts continue to be made. Targeted therapies are in variable phases of clinical development (Table 5 [16]). Some are surmised to lead to meaningful patient outcomes. A very promising novel agent is entinostat, which has done impressively well in clinical trials for the treatment of estrogen receptor-positive/HER2-negative advanced breast cancer. Entinostat belongs to the class of drugs known as HDAC inhibitors, which work through epigenetic modifications.

**Table 5:** Evolving novel agents [16].

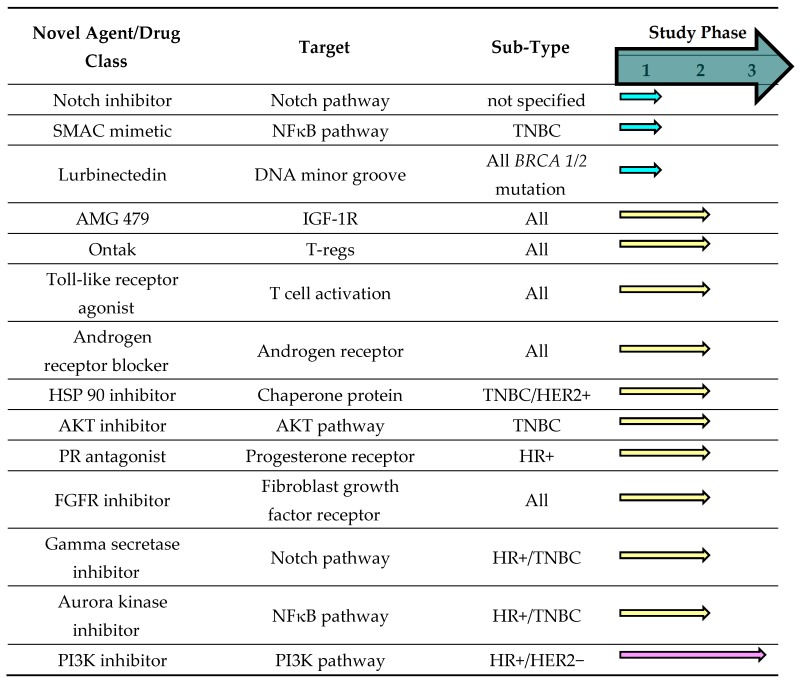

Abbreviations: ER+ = estrogen receptor positive; HR+ = hormone receptor positive; TNBC = triple negative breast cancer, SMAC = second mitochondrial-derived activator of caspases, PI3K = phosphoinositide 3-kinase, HER2- = human epidermal growth factor receptor negative, NFκB pathway = nuclear factor kappa-light-chain-enhancer of activated B cells, FGFR= fibroblast growth factor receptor 1, AMG 479 = unnamed novel agent, AKT = abbreviation for proto-oncogene also known as protein kinase B or PKB, HSP 90 = heat shock protein 90, PR = progesterone receptor, IGF-1R = insulin-like growth factor receptor 1.

**Figure 2:**
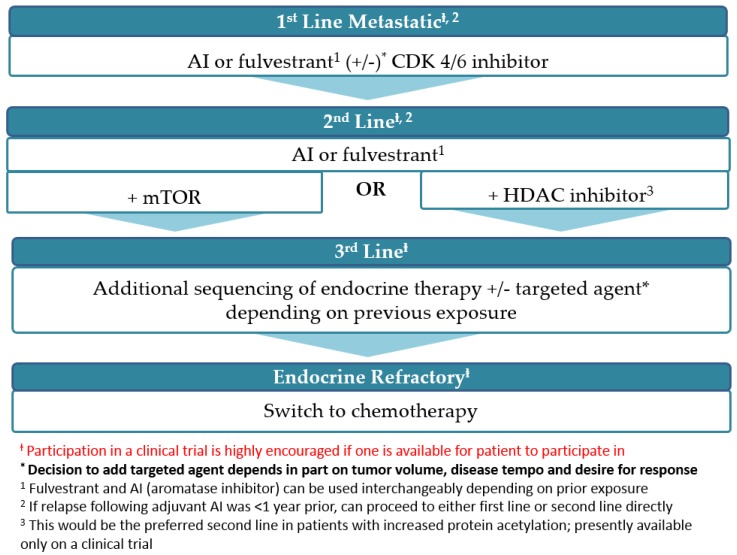
Proposed sequencing strategy for postmenopausal patients with ER+/HER2− advanced breast cancer.

Epigenetics are processes that modify transcription of DNA without altering its sequence. Epigenetic modifications such as histone acetylation/deacetylation can activate or silences genes. Through epigenetic changes, transcription of the ER gene stops, making inhibition of tumor cell growth more difficult. Histone deacetylase (HDAC) inhibitors can resensitize hormone resistant breast cancer cells by reactivating gene transcription, making tumor cells more sensitive to hormone therapy [35]. This biologic hypothesis was tested clinically; the phase II ENCORE 301 trial brought entinostat to the forefront as the most impressive HDAC inhibitor thus far [36]. Entinostat, an oral HDAC inhibitor given once weekly was tested in combination with exemestane in patients whose tumors progressed on an AI in the aforementioned trial. PFS was modest at 4.3 months with entinostat plus exemestane *vs.* 2.3 months with exemestane plus placebo. However, there was an impressive median OS advantage of 8 months with the addition of entinostat to exemestane (19.8 *vs.* 28.1 months, HR 0.59; *p* = 0.036). Entinostat was well tolerated with neutropenia and fatigue as the most common side effects. Importantly, acetylation status in peripheral blood mononuclear cells (PBMC) seemed to identify which patients derive the most benefit from HDAC inhibition. Median PFS for entinostat in a subset of patients with increased protein acetylation was 8.5 months *vs.* 2.8 months in non-acetylators [36]. Entinostat was given “Breakthrough Therapy” designation status by the FDA based on the results of ENCORE 301 trial. An ECOG-led phase III trial (E2112) of entinostat in combination with exemestane in the same disease setting is ongoing. This study will also assess acetylation status in PBMC as a predictive biomarker for response to the drug. At this time, guidelines do not recommend sequencing strategies for endocrine therapy in the metastatic setting for HR+ disease. However, given evolving data, it will not be surprising if sequencing recommendations will be made in the near future to reflect the positive results of trials using targeted agents such as palbociclib and everolimus and maybe even entinostat. A proposed recommendation is depicted in Figure 2.

Signaling pathways are not often straightforward but are complex with convergence, divergence and positive/negative feedback loops. There is a complicated interplay between downstream effectors after a single receptor is blocked. Moving forward, vigilance will be needed to be certain that targeting an alteration in the signal transduction pathway of a tumor does not inadvertently activate another pathway or trigger a feedback loop that ultimately leads to more aggressive behavior of the tumor. Fortunately, advances in molecular techniques and high-throughput technologies make it possible to interrogate the dynamic activities of multiple parallel signal transduction pathways. Such tools will hopefully provide investigators a rational way of developing, combining or sequencing targeted agents to improve responses and overcome resistance that end in improving overall outcomes for patients. Furthermore, the ability to predict who will respond to a particular targeted therapy as well as who is particularly vulnerable to its unique toxicities will be increasingly important in the era of precision medicine. Developing predictive biomarkers along with the targeted therapy is crucial. This can be quite challenging since tumor samples are often required and re-biopsy maybe difficult especially if lesions are located in places that are not readily accessible. But the greater impediment is the inherent biology of tumor cells; they are heterogeneous and may change over time, so that a biopsy from one lesion may not represent the entire tumor burden or what would turn out to be the most problematic tumor cells over time. Knowledge of which are the relevant pathways that are activated in the dominant tumor cell population in real time will become extremely important. Functional imaging with “liquid biopsies” plus advances in multiplex assays and nanotechnology, along with all the other scientific advances in the last century provide the means to accomplish this great task. For the clinician, recommending the most appropriate therapy in the era of intrinsic subtyping of breast cancer can at times be perplexing and it can only become more complex as we move further into molecular classification of breast cancer. Selecting the most efficacious therapy for every patient without the appropriate biomarker(s) is only “guess work” and there is no personalized medicine in that.

## 7. Conclusions

Our understanding of breast cancer biology has expanded exponentially since the turn of the millennia. We now understand that breast cancer is not one disease, but rather a heterogeneous group of diseases with distinct responses to treatment. The current subtyping by hormone receptor and HER2 status does not fully capture the variable behavior of breast cancers *in vivo*; molecular classification is providing better insight into this diversity. Exciting translational research has been paramount in identifying drugable alterations in important signal transduction pathways found in malignant cells that may potentially lead to important targeted agent(s) for breast cancer. Indeed, as our understanding of cancer biology improves, more targeted treatments become available. For breast cancer, providing treatment options for all but the sickest of patients is now achievable, but having a cure for everyone continues to be an elusive goal. In the era of precision medicine, developing targeted agents is simply not enough; it must go hand in hand with the development of an appropriate predictive biomarker if we are to maximize the promise of personalized medicine for our patients. The task is daunting but tremendous technological advances are being made which will lend the tools to undertake this challenge. It is imperative that action is taken, for the price of inaction to patients is great and the cost to society immense.
